# Socioeconomic status, oral health and dental disease in Australia, Canada, New Zealand and the United States

**DOI:** 10.1186/s12903-018-0630-3

**Published:** 2018-10-26

**Authors:** Gloria C. Mejia, Hawazin W. Elani, Sam Harper, W. Murray Thomson, Xiangqun Ju, Ichiro Kawachi, Jay S. Kaufman, Lisa M. Jamieson

**Affiliations:** 1 Australian Research Centre for Population Oral Health, Adelaide Dental School, The University of Adelaide, Adelaide, SA 5005 Australia; 2 Harvard School of Dental Medicine, Harvard University, Boston, MA USA; 3 Department of Epidemiology, Biostatistics & Occupational Health, McGill University, Montreal, Quebec H3A 1A2 Canada; 4 Sir John Walsh Research Institute, Faculty of Dentistry, The University of Otago, Dunedin, New Zealand; 5 Social and Behavioral Sciences, Harvard T.H. Chan School of Public Health, Boston, MA 02115 USA

**Keywords:** Socioeconomic factors, Dental caries, Self-report, Oral health

## Abstract

**Background:**

Socioeconomic inequalities are associated with oral health status, either subjectively (self-rated oral health) or objectively (clinically-diagnosed dental diseases). The aim of this study is to compare the magnitude of socioeconomic inequality in oral health and dental disease among adults in Australia, Canada, New Zealand and the United States (US).

**Methods:**

Nationally-representative survey examination data were used to calculate adjusted absolute differences (AD) in prevalence of untreated decay and fair/poor self-rated oral health (SROH) in income and education. We pooled age- and gender-adjusted inequality estimates using random effects meta-analysis.

**Results:**

New Zealand demonstrated the highest adjusted estimate for untreated decay; the US showed the highest adjusted prevalence of fair/poor SROH. The meta-analysis showed little heterogeneity across countries for the prevalence of decayed teeth; the pooled ADs were 19.7 (95% CI = 16.7–22.7) and 12.0 (95% CI = 8.4–15.7) between highest and lowest education and income groups, respectively. There was heterogeneity in the mean number of decayed teeth and in fair/poor SROH. New Zealand had the widest inequality in decay (education AD = 0.8; 95% CI = 0.4–1.2; income AD = 1.0; 95% CI = 0.5–1.5) and the US the widest inequality in fair/poor SROH (education AD = 40.4; 95% CI = 35.2–45.5; income AD = 20.5; 95% CI = 13.0–27.9).

**Conclusions:**

The differences in estimates, and variation in the magnitude of inequality, suggest the need for further examining socio-cultural and contextual determinants of oral health and dental disease in both the included and other countries.

## Background

Socioeconomic status has long held interest for its effect on general and oral health. Most evidence indicates that socioeconomic inequalities are associated with oral health status, whether subjectively (self-rated oral health) or objectively (clinically-diagnosed dental diseases) determined [1–4]. Monitoring social inequalities in oral health is important to provide information on population differences in oral health care needs, preventive practices and oral health system priorities.

Previous studies have demonstrated that socioeconomic position is negatively associated with oral health and dental disease [3, 5], which means the higher the socioeconomic position, the better the perception of oral health and the less experience of clinically-diagnosed dental diseases. Education and income are the most common and relevant indicators used in epidemiology for socioeconomic status measurement [3–7]. Oral health, as a significant constituent of general health, relies on subjective perceptions, whereas disease measurement uses objective clinical indicators [1, 2].

Most previous studies estimated the association between socioeconomic and oral health status based on national surveys or on a specific population [5, 7–10]. Population determinants of health and disease are more likely to vary across countries than within countries, but it is impossible to generalize the strength and direction of associations across populations and time [11]. Therefore, a global approach is considered fundamental to ‘public health epidemiology’ because it allows identification of international patterns that lead to hypothesis generation, essential to scientific progress [11]. In addition, these studies generally estimated the association by using only one socioeconomic factor with clinical indicators of dental disease. Few studies have tackled both subjective (health) and objective or normative (disease) aspects [12]. Some have focused on low to middle income countries, with few cross-national comparisons [13–18]. Hence, the aim of this paper is to compare the magnitude of socioeconomic inequality in oral health and dental disease using representative datasets of adults in Australia, Canada, New Zealand and the United States.

## Methods

Comparable high-income countries with dental health care delivery for the adult population based largely on fee-for-service [19, 20] were selected on the availability of nationally-representative survey examination data within a 5 year timeframe. The sources of data were: (1) Australia’s National Survey of Adult Oral Health (NSAOH), conducted between 2004 and 2006 [21]; (2) the Canadian Health Measures Survey (CHMS) that was conducted between 2007 and 2009 [22]; (3) the New Zealand Oral Health Survey (NZOHS) that was conducted from February to December 2009 [23] and; (4) the 2003–2004 module of the US National Health and Nutrition Examination Survey (NHANES) [24]. All surveys included a comprehensive oral examination and detailed demographic and socioeconomic position data.

The NSAOH used a three-stage, stratified clustered design, with 14,123 adults aged 15 years and older taking part in a telephone interview. Of these, 5,505 respondents were invited for, and accepted, a dental examination [21]. The CHMS used a multi-stage stratified sampling design to interview and examine a total of 5,586 participants, including both children and adults [22]. The NZOHS examined 3,196 children and adults. The study base were participants in the previous New Zealand 2006/2007 health survey who agreed to be contacted for future surveys; this second survey was still found to be representative [23]. NHANES, a stratified multistage probability sample of the civilian non-institutionalized population of the US, examined 7,072 people [24].

The response rates for each survey were 49.0% (the interview participation rate) and 43.7% (the examination rate) (NSAOH) [21]; 69.9% of the selected households and among households, 88.3% and 84.9% of individuals (questionnaire and clinic component, respectively) (CHMS) [22], 41.0% (NZOHS) and 79% (interview) and 76% (examination) (NHANES) [25].

In this study, health was captured through the variable self-rated oral health, an indicator of subjective oral health status. In the NSAOH, the self-rated question read: “How would you rate your own dental health?” In the CHMS, the question used was “In general, would you say the health of your mouth is…” The NZOHS asked “How would you describe the health of your teeth or mouth?” In NHANES, the question was “How would you describe the condition of your teeth?” All surveys used the following ordinal response options: ‘Excellent’, ‘Very good’, ‘Good’, ‘Fair’ or ‘Poor’. The responses were dichotomized into ‘excellent, very good or good’ and ‘fair or poor’. Disease was assessed through clinical examination by registered and calibrated dental examiners by using a standard oral epidemiological method /the examination protocol - the U.S. National Institute of Dental Research (National Institute of Dental Research 1987) [26], as untreated tooth decay (% DT > 0) and the mean number of decayed teeth (mean DT). All analyses were based on 28 teeth, excluding third molars.

We used education and income as measures of socioeconomic position. Education was grouped into 4 comparable categories across the surveys (primary, secondary, post-secondary and University). We grouped income categories for each country by quantiles into equal thirds (low, medium, high). However, when converting the categories of income from the survey into tertiles, the resulting proportions were not exactly equal because of prior categorization in the original data collected in each survey.

We limited the analysis to adults aged 25 years and older in order to have a more stable measure of final educational attainment. We calculated absolute differences in prevalence (AD) to examine socioeconomic inequalities and we estimated pooled measures of inequality estimates using random effects meta-analysis.

All analyses were age and gender adjusted to the average covariate distribution of the four surveys combined. In addition, to make population inferences, we utilized survey weights to account for individual probabilities of selection and complex survey designs [21–24]. We used Stata statistical software (version 13.1) for all analyses [27].

## Results

The combined study sample included 14,960 participants, of whom 33.9% were from Australia, 21.9% from Canada, 13.6% from New Zealand and 30.5% from the United States. Table 1 indicates that, across all countries, a slightly higher proportion of females were represented, with the mean population age ranging between 47.9 years for Canada and 49.5 years for Australia. In Australia, a greater proportion of individuals had a University educational level; in Canada, New Zealand and the United States, there was a greater proportion with post-secondary education. Australia also had the highest proportion of individuals with only primary education, and Canada the lowest.

**Table 1:** Socio-demographic and outcome characteristics

Variables	Australia	Canada	New Zealand	United States
	National Survey of Adult Oral Health	Canadian Health Measures Survey^a^	New Zealand Oral Health Survey	National Health and Nutrition Examination Survey
	2004–2006	2007–2009	2009	2003–2004
	*N* = 5,073	*N* = 3,278	*N* = 2,041	*N* = 4,568
	N (%)^b^	N (%)^b^	N (%)^b^	N (%)^b^
Gender				
Male	2,016 (49.8)	(49.1)	793 (48.2)	2,200 (48.0)
Female	3,057 (50.2)	(50.9)	1,248 (51.9)	2,368 (52.0)
Education				
Primary	1,196 (22.8)	(12.8)	467 (19.8)	1,366 (18.6)
Secondary	478 (10.9)	(17.6)	307 (16.4)	1,134 (26.9)
Post-Secondary	1,521 (32.0)	(42.1)	800 (40.0)	1,189 (30.4)
Tertiary	1,634 (34.4)	(27.5)	444 (23.3)	865 (24.2)
Income				
Low	2,189 (37.4)	(6.3)	776 (24.7)	2,075 (36.1)
Medium	1,590 (35.0)	(36.0)	592 (29.0)	1,157 (30.2)
High	1,069 (27.6)	(57.7)	728 (46.4)	1,060 (33.8)
Mean age (years)	49.5 ± 14.7	47.9 ± 12.4	48.9 ± 13.0	48.9 ± 13.0

NB: all numbers are based on individuals aged 25 years or older

^a^Due to reasons of confidentiality, the only estimates available for Canada are weighted proportions (i.e. not N)

^b^Weighted proportions

Table 2 shows differences in prevalence and mean estimates among countries; for example, the prevalence of decayed teeth for highly educated New Zealanders was equal to that of the lowest educated group in the United States. The same was observed in the mean number of teeth with untreated decay. Downward gradients by educational level and income within countries favored the more socially advantaged socioeconomic groups. Australia showed a clear gradient in the adjusted estimate for the two disease measures (% DT > 0 and Mean DT) but it is less obvious for fair/poor self-rated oral health. Canada presents a gradient in the proportion of individuals with at least one untreated decayed tooth. New Zealand shows educational gradients in the proportion with untreated decay and fair/poor self-rated oral health. The apparent inconsistency in educational gradients for Canada and New Zealand in disease severity (mean DT) was minor and is likely explained by sampling variability. The United States consistently showed gradients that favor the most highly educated. By income, all countries present gradients for all measures in which lower income groups are more heavily burdened with poorer oral health.

**Table 2:** Adjusted estimates and adjusted absolute difference (AD) for multiple oral health outcomes

	% DT > 0		Mean DT		% Fair/poor self-rated oral health	
	Adjusted estimate	AD	Adjusted estimate	AD	Adjusted estimate	AD
	(95% CI)	(95% CI)	(95% CI)	(95% CI)	(95% CI)	(95% CI)
Australia^a^						
Education						
Primary	32.7 (28.1, 37.2)	18.4 (13.1, 23.7)	0.8 (0.7, 1.0)	0.6 (0.4, 0.8)	24.5 (22.4, 26.6)	10.9 (8.4, 13.3)
Secondary	28.6 (22.7, 34.6)	14.4 (7.7, 21.0)	0.5 (0.4, 0.7)	0.3 (0.2, 0.5)	16.3 (13.4, 19.1)	2.7 (−0.4, 5.7)
Post-secondary	23.3 (19.9, 26.6)	9.0 (4.9, 13.1)	0.5 (0.4, 0.6)	0.3 (0.2, 0.4)	17.7 (16.1, 19.4)	4.1 (2.0, 6.3)
University	14.3 (11.8, 16.8)	Ref	0.2 (0.2, 0.3)	Ref	13.6 (12.1, 15.1)	Ref
Income						
Low	27.7 (24.3, 31.0)	11.9 (6.8, 17.0)	0.6 (0.5, 0.7)	0.4 (0.2, 0.5)	25.3 (23.2, 27.4)	13.9 (11.1, 16.8)
Medium	22.5 (19.5, 25.5)	6.8 (2.1, 11.4)	0.4 (0.4, 0.5)	0.2 (0.1, 0.3)	14.4 (13.0, 15.9)	3.1 (0.9, 5.2)
High	15.8 (12.3, 19.3)	Ref	0.3 (0.2, 0.3)	Ref	11.4 (9.6, 13.1)	Ref
Canada^b^						
Education						
Primary	32.3 (25.2, 39.5)	22.1 (14.2, 30.0)	0.9 (0.6, 1.1)	0.7 (0.4, 0.9)	23.7 (17.7, 29.6)	13.0 (6.2, 19.7)
Secondary	25.4 (19.9, 31.0)	15.2 (8.8, 21.6)	0.9 (0.6, 1.3)	0.7 (0.4, 1.1)	24.5 (18.7, 30.3)	13.8 (7.3, 20.4)
Post-secondary	18.0 (15.0, 21.1)	7.8 (3.7, 12.0)	0.4 (0.4, 0.5)	0.2 (0.1, 0.3)	13.1 (10.6, 15.5)	2.4 (−1.3, 6.1)
University	10.2 (7.3, 13.2)	Ref	0.2 (0.1, 0.3)	Ref	10.7 (7.9,13.5)	Ref
Income						
Low	31.1 (22.5, 39.8)	16.7 (7.5, 25.9)	0.9 (0.6, 1.2)	0.6 (0.3, 1.0)	28.4 (20.7, 36.0)	16.9 (8.9, 24.9)
Medium	22.4 (18.5, 26.4)	8.0 (3.0, 12.9)	0.7 (0.5, 0.8)	0.4 (0.2, 0.5)	19.7 (16.3, 23.1)	8.3 (4.1, 12.5)
High	14.5 (11.9, 17.1)	Ref	0.3 (0.2, 0.4)	Ref	11.4 (9.2, 13.7)	Ref
New Zealand^c^						
Education						
Primary	46.5 (37.6, 55.5)	17.7 (6.6, 28.7)	1.4 (1.0, 1.7)	0.8 (0.4, 1.2)	41.0 (32.0, 49.9)	17.6 (7.1, 28.1)
Secondary	42.9 (36.8, 49.0)	14.1 (5.1, 23.1)	0.9 (0.8, 1.1)	0.4 (0.1, 0.6)	30.1 (24.4, 35.8)	6.7 (−1.3, 14.7)
Post-secondary	35.6 (30.9, 40.3)	6.8 (−1.3, 14.8)	1.1 (0.9, 1.3)	0.5 (0.2, 0.8)	27.5 (23.3, 31.8)	4.2 (−2.9, 11.2)
University	28.8 (22.4, 35.3)	Ref	0.6 (0.4, 0.7)	Ref	23.4 (17.9, 28.9)	Ref
Income						
Low	43.4 (36.0, 50.8)	17.5 (8.0, 26.9)	1.5 (1.1, 2.0)	1.0 (0.5, 1.5)	38.3 (31.1, 45.5)	18.8 (9.8, 27.8)
Medium	40.3 (33.2, 47.4)	14.3 (5.4, 23.3)	0.9 (0.7, 1.1)	0.4 (0.2, 0.6)	31.4 (24.6, 38.2)	11.9 (3.7, 20.1)
High	26.0 (20.5, 31.4)	Ref	0.5 (0.4, 0.7)	Ref	19.6 (14.8, 24.3)	Ref
United States^d^						
Education						
Primary	28.7 (24.1, 33.3)	20.1 (15.8, 24.5)	0.6 (0.5, 0.7)	0.4 (0.3, 0.5)	64.5 (61.1 67.9)	40.4 (35.2, 45.5)
Secondary	19.0 (13.8, 24.2)	10.4 (4.7, 16.2)	0.4 (0.3, 0.5)	0.3 (0.1, 0.4)	51.7 (47.4, 56.0)	27.5 (22.6, 32.5)
Post-secondary	16.3 (12.7, 19.9)	7.7 (3.6, 11.8)	0.3 (0.2, 0.4)	0.2 (0.1, 0.2)	45.0 (41.1, 48.9)	20.8 (16.3, 25.4)
University	8.6 (5.5, 11.7)	Ref	0.2 (0.1, 0.2)	Ref	24.1 (20.1, 28.2)	Ref
Income						
Low	21.1 (17.4, 24.8)	8.9 (4.8, 13.1)	0.4 (0.3, 0.5)	0.2 (0.1, 0.4)	54.0 (50.0, 58.1)	20.5 (13.0, 28.0)
Medium	15.6 (11.3, 20.0)	3.4 (−2.2, 9.0)	0.3 (0.2, 0.4)	0.1 (−0.1, 0.2)	43.5 (38.6, 48.4)	10.0 (3.8, 16.2)
High	12.2 (8.3, 16.2)	Ref	0.2 (0.1, 0.3)	Ref	33.5 (28.7, 38.4)	Ref

NB: Data based on ages 25 years and older. Education adjusted for age and gender and Income adjusted for age, gender and education

^a^National Survey of Adult Oral Health, 2004–2006

^b^Canadian Health Measures Survey, 2007–2009

^c^New Zealand Oral Health Survey, 2009

^d^National Health and Nutrition Examination Survey, 2003–2004

As indicated by the adjusted absolute differences in Table 2, the greatest absolute inequalities between the extreme levels of education (Primary versus University) were in Canada for the proportion of individuals with untreated decay (AD = 22.1), in New Zealand for the mean number of untreated decayed teeth (AD = 0.8), and in the United States for fair/poor self-rated oral health (AD = 40.4). Also, shown in Table 2, the greatest absolute inequality in outcomes between extreme levels of income (Low versus High) is in New Zealand for the proportion with untreated decay (AD = 17.5) and the mean number of untreated decayed teeth (AD = 0.99), whereas, for fair or poor self-rated oral health, the greatest gap is in the United States (AD = 20.5).

**Fig. 1:**
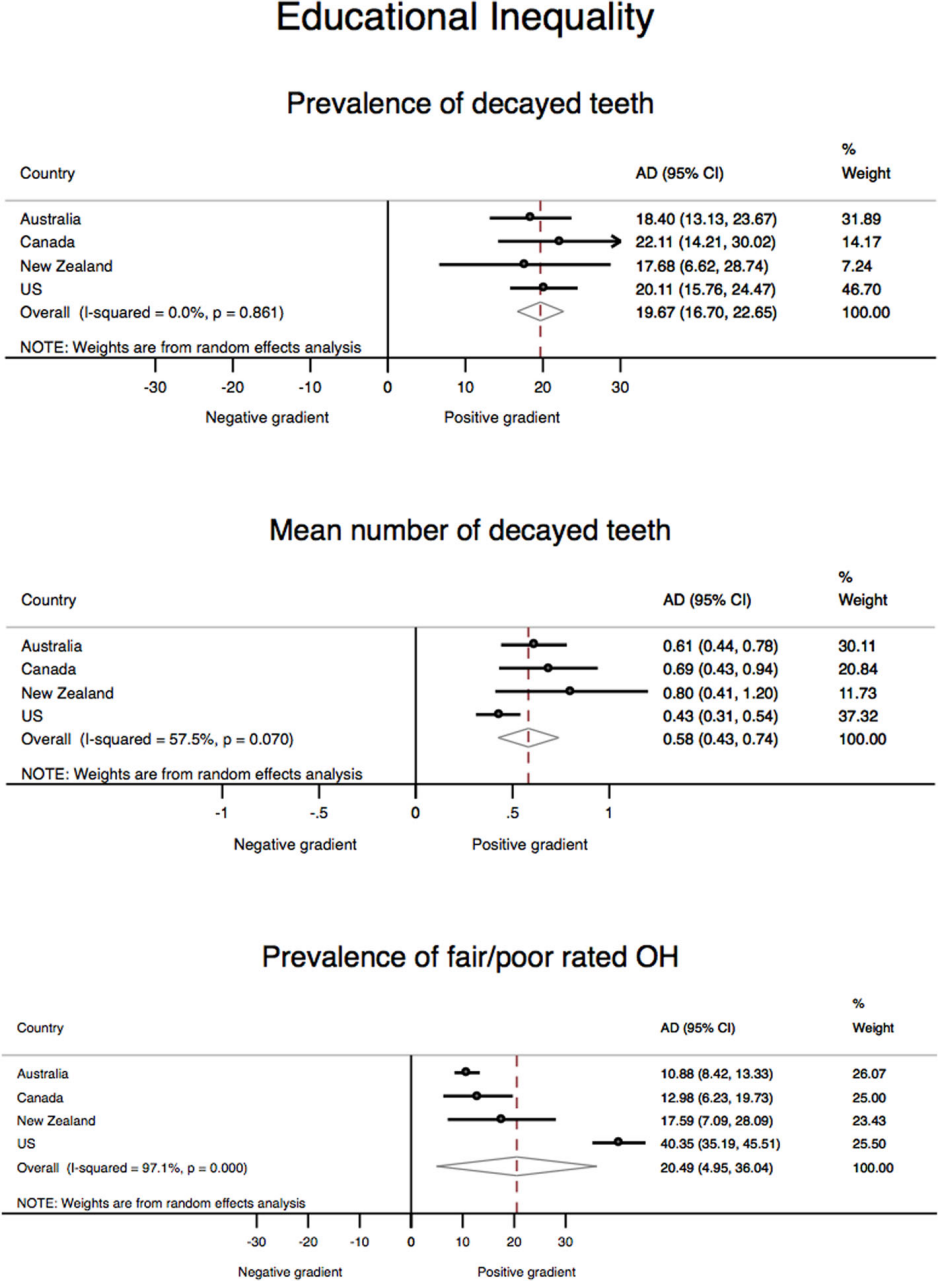
Meta-analysis estimates for educational inequality

Figure 1 presents meta-analysis estimates for educational inequality. The findings on educational inequality for the proportion of individuals with at least one tooth with untreated decay indicate that all variability in the effect sizes is attributable to sampling error (I^2^ = 0.0%); results for this measure may be considered to be essentially homogenous, with a pooled adjusted AD of 19.7. There was moderate heterogeneity (I^2^ = 57.5%) for the mean number of untreated decayed teeth; that is, roughly half of the variability was among countries and half of the variability was within countries. New Zealand had the widest absolute socioeconomic inequality (AD = 0.8) and the United States had the narrowest (AD = 0.4). For fair or poor self-rated oral health, the United States had the widest inequality gap (AD = 40.4) and Australia the narrowest (AD = 10.9) with almost all of the variation occurring across countries (I^2^ = 97.1%).

**Fig. 2:**
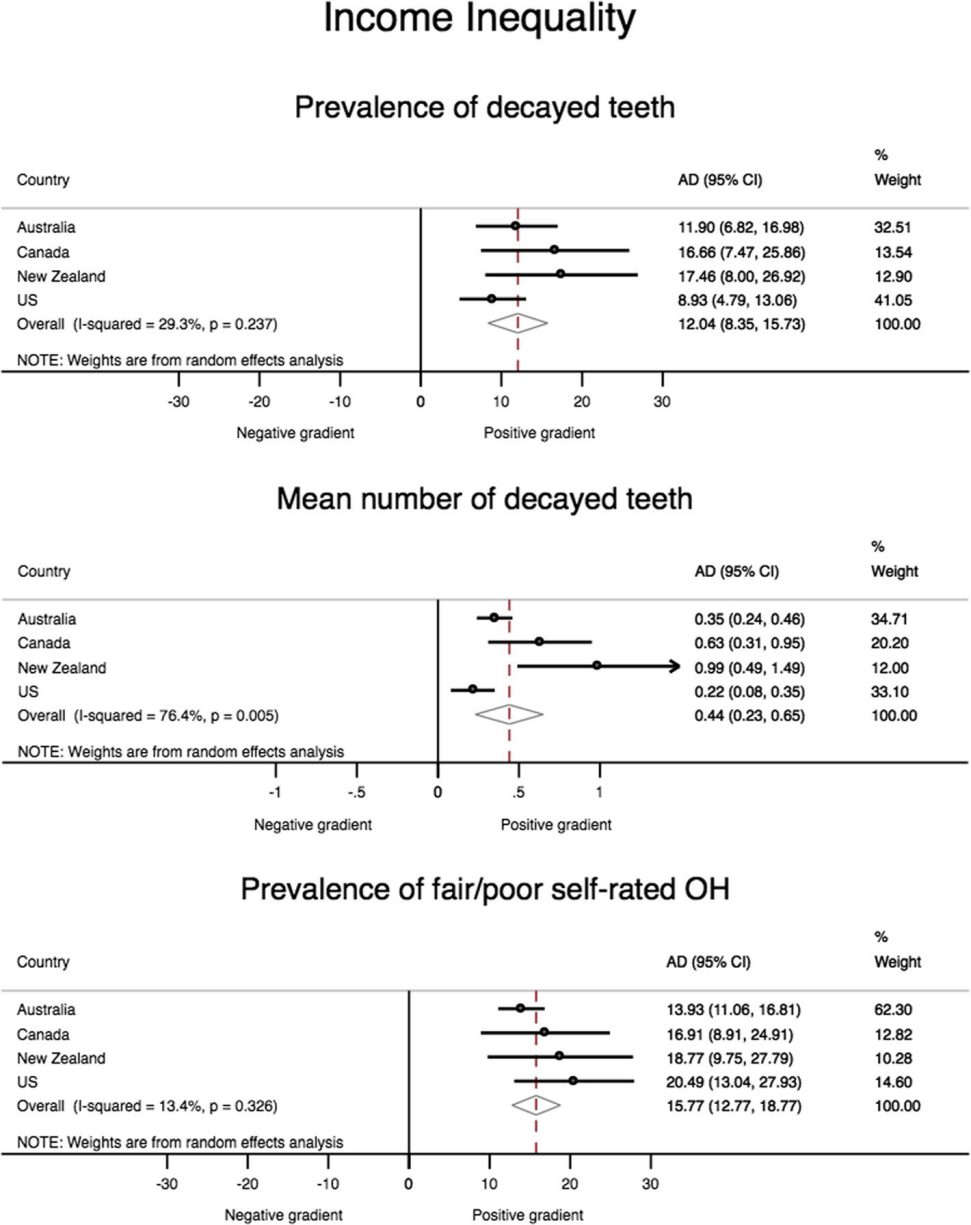
Meta-analysis for income inequality

The meta-analysis for income inequality (Fig. 2) indicates modest heterogeneity across countries in the pooled estimate for the prevalence of untreated decayed teeth (I^2^ = 29.3%), with New Zealand having the widest gap and the United States the narrowest (AD = 17.5 and 8.9, respectively). The other measure of dental disease ─ the mean number of teeth with untreated decay ─ showed more profound heterogeneity of effect estimates (I^2^ = 76.4%). Again, New Zealand presented the greatest magnitude of absolute inequality (AD = 0.99), translating into a clinical difference of one tooth, and the United States presented the lowest magnitude of effect (AD = 0.22). There was low heterogeneity in the measure of fair/poor self-rated oral health, with a pooled adjusted absolute difference of 15.8 percentage points, ranging from 13.9 for Australia to 20.5 for the United States.

## Discussion

The findings demonstrate socioeconomic inequality in self-rated oral health and untreated dental caries among adults in Australia, Canada, New Zealand and the United States, yet they also highlight some important differences across countries. While New Zealand had the highest absolute inequality in measures of disease, the United States had the highest gaps in perceptions of oral health.

Measures of health status based solely on the objective assessment of pathological abnormality do not include non-biological aspects of health such as the mental and social wellbeing of individuals. We represented disease through normative clinical measures of untreated tooth decay and the magnitude or extent of the disease through the mean number of decayed teeth. To measure oral health, we used self-rated health, considered as “the most feasible, most inclusive and most informative measure of health status” [28].

Interestingly, our findings indicate that New Zealand had greater disease and wider socioeconomic gaps in the proportion and mean number of untreated decay than the other countries, despite having arguably the most comprehensive, wide ranging and free public dental service in the world, that is available to all aged below 18 years (through the School Dental Service). A possible explanation may lie in New Zealand not having a means-tested public dental service for low-income adults (those aged 18+ years), whereas such services are available in Canada, Australia and, to some extent, the United States.

Our study did not examine the effects of other contributing factors such as water fluoridation. It is estimated that 79% of the Australian population, 53% of the New Zealand population, 42% of Canadian population and 60% of the US population is supplied with artificially fluoridated water [29]. Water fluoridation has been regarded as the most effective way to reduce the prevalence and severity of caries, as well as socioeconomic disparities in its occurrence [30]. Although a side-effect of water fluoridation is mild fluorosis of enamel, manifesting a slightly more opaque enamel that is generally perceived by lay people as being aesthetically better, with concomitant effects on their self-rated oral health [31].

The United States had the most unfavorable indicators of oral health, in terms of self-ratings, which is in sharp contrast with self-ratings of general health, in which Americans perform relatively well [32]. In global health measures, the intrinsic value individuals assign to health is driven by a multitude of factors including socio-cultural environments and personal experiences [33].

Dissatisfaction with dental appearance is associated with tooth alignment and crowding, fractures in anterior teeth, and discrepancies in tooth shade [34–36]. It relates respectively to orthodontic treatment, aesthetic restorations and tooth bleaching [34, 35, 37]. Oro-facial aesthetics and appearance have been shown to be associated with self-ratings of oral health in diverse population samples [36, 38–41]. It is possible that the contemporary emphasis on dental aesthetics (such as tooth whitening) contributes to a general dissatisfaction in dental appearance and hence poorer self-rated oral health, whereas the same does not occur for general health.

Differences in reporting may arise from cultural perceptions of health, differences in health expectations and adaptability to ill-health, but also from the way in which the ordinal scale is understood by different individuals and how they weigh the different factors involved in the global measure [42, 43]. In this study, the wording of the SROH question varied slightly among the countries, with NSAOH and NHANES asking specifically about dental health/teeth, Canada framed the question in terms of health of the mouth and New Zealand asked for the health of both the teeth and mouth. Also noteworthy is that the United States asked about the ‘condition’ of the teeth, whereas all other countries framed the question around ‘health’, which could influence how the question is interpreted and may aid in explaining the large difference between the United States and the other countries. A limitation of the study was the inability to measure the extent to which the differences in terminology influenced the findings.

Differences in self-reports may be explained in terms of optimism, such as the ability among older people to adapt to slow declining health, and higher expectations when more socially advantaged groups, for example, report poorer health states [44]. Rousseau and colleagues [45] reported as such, arguing that the complete loss of all teeth is considered by middle-class people to be far more catastrophic than it is by working-class people, because of differing social norms. It is also possible that the frame of reference through which societies in a given country view disease differs; for example, Australians had the lowest levels of self-rated fair or poor health yet their levels of disease were as high as or higher than disease in the United States. Even subtle differences in subjective ratings point towards cultural, social and psychosocial influences on oral health [46]. Given the cultural and context-specific nature of self-rated health, our findings cannot necessarily be generalized to countries beyond those included in the analysis, and caution needs to be taken when making international comparisons [28, 33].

The study explored two socioeconomic indicators to draw a clear picture of social inequalities. Education has the potential to translate into employment opportunities, receptiveness to health messages and the ability to navigate health care systems, as well as representing values, beliefs, and attitudes. It captures the long-term effects of early life conditions and adult resources on health [47]. Income, which measures material resources and living standards, has a cumulative effect over the life course yet is dynamic in the short-term and may be prone to reverse causality if deteriorating health contributes to changes in income [47].

Whereas educational gradients in oral health and disease show some inconsistencies among countries, income shows consistently clear gradients across all countries. In terms of dental disease, this reflects the ability to access oral health care, favoring populations with higher income. In terms of perceived oral health, those with lower incomes reported lower self-ratings. If this general dissatisfaction were to lead to lower self-ratings than warranted by ‘objective’ health, and higher social groups were to systematically report better health than justified, such differences could lead to overestimates of health inequities [43]. Our study did not explore such possibilities at the individual level, but, on average, socially advantaged groups had better oral health, indicating that such overestimation is unlikely.

The limitations of the study were:

(1) there was no overlap of time period for all four surveys, although it is unlikely inequality estimates would differ systematically as major changes in chronic dental diseases are not expected within short time frames; and (2) Missing data for each survey could affect the findings; however, analyses of bias due to survey non-response was carried out independently, at least in the NSAOH, [21] indicating estimates are unlikely to be affected by systematic error. In addition we used weighted data to account for sampling probabilities and adjusted for age and gender.

An important next step is to compare socio-cultural and health system characteristics that shape disease and health status measures among different countries in order to have a better understanding of the roles these factors and other social determinants play in population oral health.

## Conclusion

Our findings demonstrate differences in oral health and dental disease experience across income and education groups, with socioeconomic gradients for both clinically determined and self-reported indicators. Individuals from lower income and education groups consistently experienced higher burdens of untreated dental decay and poorer self-rated oral health. Differences in outcome estimates within countries also indicate conceptual differences between health and disease. The variation in the magnitude of inequality across countries suggests the need for further understanding socio-cultural and contextual determinants of oral health and dental disease.

## Abbreviations

AD: Absolute differences
CHMS: The Canadian Health Measures Survey
DT: Decayed teeth
NHANES: The US National Health and Nutrition Examination Survey
NSAOH: Australia’s National Survey of Adult Oral Health
NZOHS: The New Zealand Oral Health Survey
SROH: Self-rated oral health
US: The United States
